# Comparison of Renal Functions Evaluated by Measured Glomerular Filtration Rate in Patients Treated With Cisplatin, Carboplatin, and Oxaliplatin

**DOI:** 10.7759/cureus.36549

**Published:** 2023-03-22

**Authors:** Rajnee Choudhary, Manoj K Bundela, Kushaal Bharang

**Affiliations:** 1 Physiology, Dr. Sampurnanand Medical College, Jodhpur, IND; 2 Physiology, Government Medical College & Hospital, Barmer, IND

**Keywords:** 99mtc-dtpa, mgfr, nephrotoxicity, platinum drugs, chemotherapy

## Abstract

Introduction: In cancer chemotherapy platinum drugs do cause damage to the normal cells and as a result, many physiological functions are derailed. Renal function as measured by measured glomerular filtration rate (mGFR) plays a large role in drug dosing on the basis of the maximum tolerated dose, which is the highest dose that may be administered without unacceptable toxicity, to maximize anticancer efficacy.

Objective: The objective of the study was to compare the toxic effect of platinum drugs on renal function as measured by mGFR in patients with malignancy and to study the difference in the magnitude of nephrotoxicity by these drugs.

Methodology: The study was conducted in the Department of Physiology in close collaboration with the Department of Radiotherapy at a tertiary care centre in Western Rajasthan, India. 150 patients suffering from different malignances undergoing treatment with cisplatin, carboplatin, and oxaliplatin were examined for their renal function as measured by mGFR using ^99m^Tc-diethylene triamine pentaacetic acid (^99m^Tc-DTPA) and were compared with 50 subjects of control group.

Results: In the cisplatin group there was a gradual fall of GFR from 85.49 ml/min/1.73sqm to 58.09ml/min/1.73sqm at cycle II. In the carboplatin group it was 84.86ml/min/1.73sqm at baseline whereas cycle II was 75.5 ml/min/1.73sqm with SD ± 16.49. mGFR fell significantly (p<0.0001) in cisplatin and carboplatin groups but not in the cohort of patients who received oxaliplatin. The GFR reduction continued from the baseline to cycle I and then cycle II in cisplatin and carboplatin groups.

Conclusion: Nephrotoxicity is a major side effect of platin drugs and further studies should be done to establish their optimal dose in relation to renal function and minimize toxicity by trying various cytoprotective agents.

## Introduction

Chemotherapy employs systemically administrated drugs that directly damage cellular DNA (and RNA). There are several cytotoxic drugs which include: (1) DNA-damaging agents, (2) antimetabolites, (3) DNA repair inhibitors, and (4) antitubulin. Among the DNA damaging agents there are two groups: (a) free radicals-alkylators e.g. cyclophosphamide, (b) DNA cross-linking platinum drugs e.g. cisplatin, carboplatin, and oxaliplatin. The dose and schedule of chemotherapy are limited by tissue tolerance, especially in those more proliferative tissues of the bone marrow and gastrointestinal tract mucosa [1]. DNA cross-linking platinum products are used as chemotherapeutic agents for a wide range of malignancies. Effective platinum-based therapies also are in place for advanced stages of non-small cell lung cancer, colorectal cancer, esophageal cancer, gastric cancer, and head and neck malignancies [2]. Cisplatin has a number of dose-limiting side effects including nephrotoxicity, myelosuppression, ototoxicity, peripheral neuropathy, and hypomagnesemia. However, despite these toxicities, cisplatin is the backbone for many chemotherapeutic regimens in an ambulatory settings. The dosage of platinum drugs is determined according to body surface area, and is often used on an outpatient basis. Oxaliplatin is licensed for the treatment of metastatic colorectal cancer in combination with ﬂuorouracil and folinic acid. It causes side effects that include gastrointestinal disturbances, ototoxicity, and myelosuppression [3].

In most studies it has been reported that platinum compounds are highly toxic to kidneys, as they are largely excreted by the kidneys. Nephrotoxicity is an important class effect of platinum agents and hence renal function assessment is of paramount importance before using them [4]. Three drugs namely cisplatin, carboplatin, and oxaliplatin are used in the treatment of cancer patients at our centre. Since the nephrotoxicity effects of these drugs are known, we planned to examine the difference in magnitude of nephrotoxicity caused by these drugs by performing renal scanning. We used technetium-99m-diethyl-triamine-penta-acetic acid (99mTc-DTPA). In the measured GFR (mGFR) method 99mTc-DTPA is injected intravenously into the patient. It emits X-rays which are picked up by a gamma camera and converted into electronic signals. These signals reflect renal functional status. This study was thus conducted with the objective of determining the change in mGFR with different cycles of these drugs.

## Materials and methods

The research was carried out at the Department of Physiology in close collaboration with the Department of Radiotherapy at Sardar Patel Medical College, Bikaner, India. Histo-pathologically confirmed 150 patients suffering from different malignancies receiving treatment with cisplatin, carboplatin, and oxaliplatin were examined for their kidney functions and were compared with control group. Institutional ethical clearance was obtained before the commencement of the study from the Institutional Research Board, Sardar Patel Medical College, Bikaner (approval no. F.76 (Acad.)SPMC/2007-08/187-A). Informed consent was taken beforehand, whole procedure and motive of the study was explained to the subjects and their relative.

A total of 150 patients were divided into three equal study groups, i.e. 50 in each group and they were planned for either cisplatin, carboplatin, or oxaliplatin-based chemotherapy. All three study groups were given three cycles of chemotherapy and examined for their renal functions and 50 healthy controls were selected from attendants of patients, fulfilling the same inclusion criteria except presence of cancer. Chemotherapy was administered to the study groups with the given protocol.

Study group I: the patients were given cisplatin

Cisplatin solution with 5-fluorouracil was administered by intravenous infusion over a period of 3-5 hours. The recommended dose of cisplatin for adults as a single agent therapy is 50-75 mg/m^2^ body surface area every 3-4 weeks. According to protocol of the drug, chemotherapy in the form of cisplatin solution with 1 gm 5-fluorouracil in 500 ml of normal saline was given on day 1 and day 2 (first cycle). The same regime was given on the 22 and 23 day (second cycle) and on the 44 and 45 day (third cycle). Pretreatment hydration and other premedication were given prior to cisplatin infusion.

Study group II: the patients were given carboplatin

It was given in doses of 400 mg/sqm body surface area by intravenous infusion over a period of 15 minutes to 1 hour. According to protocol, carboplatin solution with 1 gram 5-fluorouracil in 500 ml of normal saline was given intravenously on day 1 (first cycle), day 22 (second cycle) and day 44 (third cycle). The infusion was given with anti-emetics and other supportive treatments.

Study group III: the patients were given oxaliplatin

It was given in doses of 85 mg/m^2^ body surface area by intravenous infusion and the treatment dose was repeated in a cycle of 2 weeks. In a continuous intravenous (IV) infusion oxaliplatin in (doses of 85 mg/m^2^ body surface area) 500 ml of 5% dextrose solution was given in the first one hour, followed by 50 mg leucovorin in 500 ml normal saline was given in next 2 hours. Identical types of regimes were given on day 15 (second cycle) and day 30 (third cycle).

Inclusion criteria: Histo-pathologically confirmed, patients with adequate renal functions (i.e. normal mGFR > 60 mL/min/1.73sqm) and hematological values, age limit: 18-60 years, chemotherapy-naive patients, no other serious medical or psychiatric illness. This is in accordance with the institutional and Government of India guidelines. Women/men of reproductive age group must agree to use effective contraceptive methods.

Exclusion criteria: Patients with urogenital cancer, systemic diseases, altered renal functions (i.e. mGFR < 60 mL/min/1.73sqm), not chemotherapy-naive, pregnant and lactating women, inability to eat orally, malabsorption syndrome, patients requiring more than six weeks time to recover from side effects of drugs were excluded.

**Figure 1:**
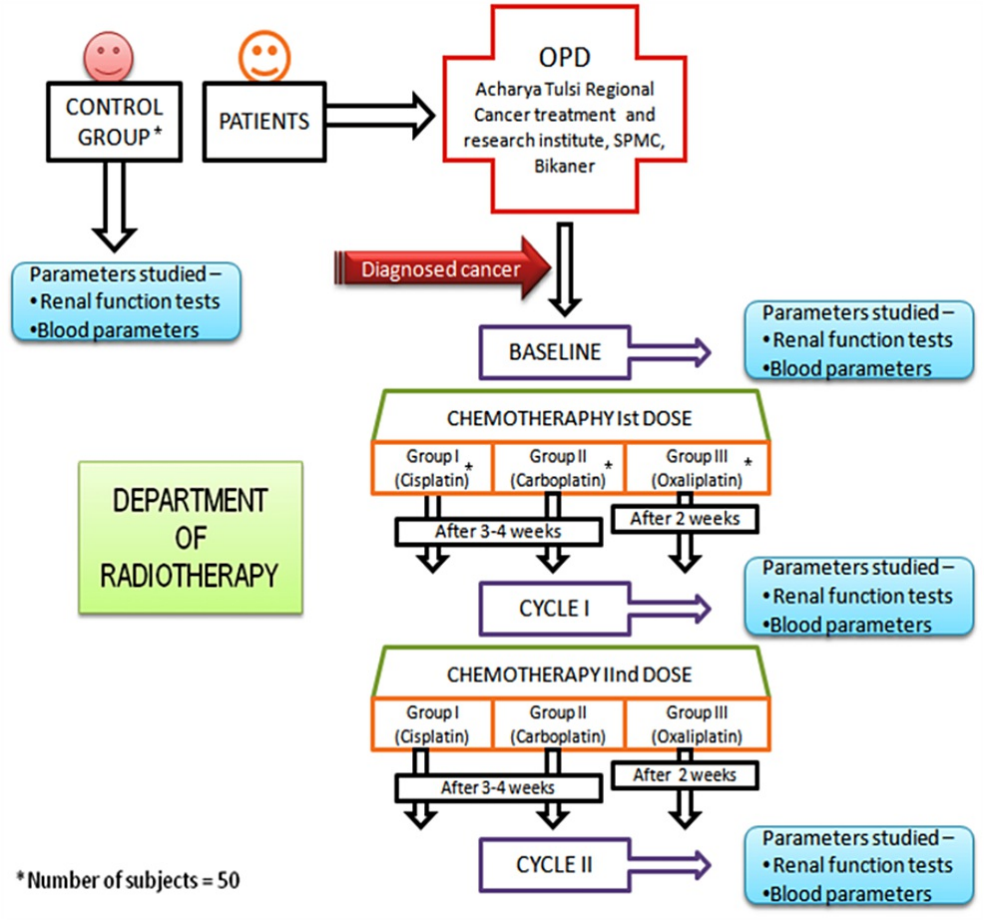
Study Design SPMC: Sardar Patel Medical College

Investigations: Relevant routine laboratory investigations like hemoglobin (Hb), total leucocyte count (TLC), platelets, complete blood count (CBC), blood urea, serum creatinine, renogram, liver function tests (LFT), X-ray chest, ultrasonography abdomen along with renal scanning using technetium-99m-diethyl-triamine-penta-acetic acid (99mTc-DTPA) were done before the starting of first, second and third cycle of treatment. as mention below in study design (Figure 1).

Measured GFR (mGFR) using urinary or plasma clearance of exogenous filtration markers is considered the gold standard for the evaluation of kidney function. mGFR measurement was calculated by using Gate’s method. Dynamic kidney renography was performed by computerized dual head spect and whole body digital gamma camera (Nuclear Spirit DH-V; MEDISO LIMITED; HQ Budapest, Hungary). After image acquisition, the patient’s weight and height were entered into the system, on which all imaging data were recorded, and the GFR was automatically calculated according to the Gate’s method. The Gate's method is most commonly used to compute the GFR in DTPA renogram. In Gate's method, the percentage renal uptake (%RU) of 99mTc DTPA is computed first. In the mGFR method, 99mTc-DTPA is injected into the patient. It emits X-rays which are picked up by a gamma camera and converted into electronic signals. These signals reflect renal functional status. 

Data collection: After obtaining the informed written consent, personal particulars like age, gender, occupation, present and past history were documented. General and systemic examination was done, vitals recorded, the outcome was documented.

Protocol of sample collection/Blinding: In this study blinding was not possible but attempts were made to minimize bias, wherever possible. Blood and urine samples were coded and stored until the final statistical analysis. No midterm analysis of the results was carried out. Care was taken to keep all four groups separate to prevent the exchange of information.

Statistical analysis

For the statistical analysis of data, appropriate statistical models were applied. Since the study was conducted in one place only, hence geographical and climatic conditions were similar in all cases. Data were managed on excel spreadsheets. The study variables were summarized by mean and standard deviation. For comparison of mean, ANOVA and student’s t-test was employed wherever applicable and probability was calculated at respective degree of freedom by SPSS software version 17.0. (SPSS Inc., Chicago, USA). 

## Results

The study comprised a total of 150 patients with cancer and 50 control subjects. Patients were divided into three groups of 50 each. Each group received one drug in three cycles. And the degree of toxicity of one particular drug with those of others was studied. In our study there were 118 males and 82 females and most of the subjects were of age group between 32 to 60 years. In gastrointestinal tract (GIT) malignancy, altogether 81 colorectal cancer patients were included primarily to avoid nutrient absorption problems which might affect hematological parameters. Similarly we included head and neck (54 patients) and thoracic cancer (15 patients) cases to maintain feeding and adequate oral hydration assuring minimum nephrotoxicity and bone marrow suppression. Along with this, during the course of treatment two patients were referred to another center and one patient left against medical advice so these patients were excluded from the study. As these three patients were excluded from study and all 150 study participants received three cycles of chemotherapy, it corresponds to 100% compliance.

Male-to-female ratio in predefine groups along with rationale was 2.8:1 in group I, 2.1:1 in group II and 1.2:1 in group III 2.8:1, while in the control group male-female ratio was 1.3:1; there were more males than females. The mean age and gender among different groups were nearly the same and there was no statistically significant difference (p>0.05) in the mean age among the four groups (Table 1).

**Table 1:** Comparison between the groups according to their age and gender

Gender	Age (years)							
	Control group (n=50)		Study group					
			Group I (n=50)		Group II (n=50)		Group III (n=50)	
	No.	Mean ± SD	No.	Mean ± SD	No.	Mean ± SD	No.	Mean ±SD
Male	29	47 ±12.63	37	47.86 ±8.93	34	51.11 ±8.27	28	46.14 ±12.10
Female	21	42.52 ±12.09	13	44.54 ±7.32	16	48.62 ±5.08	22	46.23 ±10.71
Male	t-test		0.32		1.55		0.26	
	p-value		0.75		0.12		0.79	
Female	t-test		0.54		1.89		1.06	
	p-value		0.59		0.06		0.29	

Mean value of mGFR in normal control group and cycles of study groups shows that in group I GFR mean value was 85.49±20.83 in baseline, 66.66±22.40 in cycle I and 58.09±21.86 in cycle II. We found that the mean values of cycle I and cycle II were statistically significantly lower (p<0.0001) than that of the baseline.

For group II GFR mean value of cycle II (75.55±16.49) was statistically significantly lower (p=0.001) than that of baseline (84.86±10.33). However, the mean values of GFR in group III shows no significant variations (Table 2).

**Table 2:** mGFR (ml/min/1.73sqm) in control and three study groups receiving chemotherapy in cycles n = 50 subjects in each group; BL = Baseline; CI = Cycle I; CII = Cycle II; CG = Control group; GI = Group I; GII = Group II; GIII = Group III. p<0.05 was considered statistically significant. Glomerular filtration rate (GFR ) in ml/min/1.73sqm expressed as mean±SD.

Groups (n=50)		Baseline	Cycle I	Cycle II	p-value		
		Mean ±SD	Mean ±SD	Mean ±SD	^BL vs. CI^	^BL vs. CII^	^CI vs. CII^
Control group		91.05 ±18.40	-	-	-	-	-
Group I		85.49 ±20.83	66.66 ±22.40	58.09 ±21.86	0.000	0.000	0.151
Group II		84.86 ±10.33	81.27 ±9.74	75.55 ±16.49	0.466	0.001	0.073
Group III		87.38 ±13.32	82.05 ±13.92	81.02 ±12.98	0.146	0.06	1.0
p-value	^CG vs. GI^	0.534	-	-			
	^CG vs. GII^	0.349	-	-			
	^CG vs. GIII^	1.0	-	-			
	^GI vs. GII^	1.0	0.000	0.000			
	^GI vs. GIII^	1.0	0.000	0.000			
	^GII vs. GIII^	1.0	1.0	0.361			

**Figure 2:**
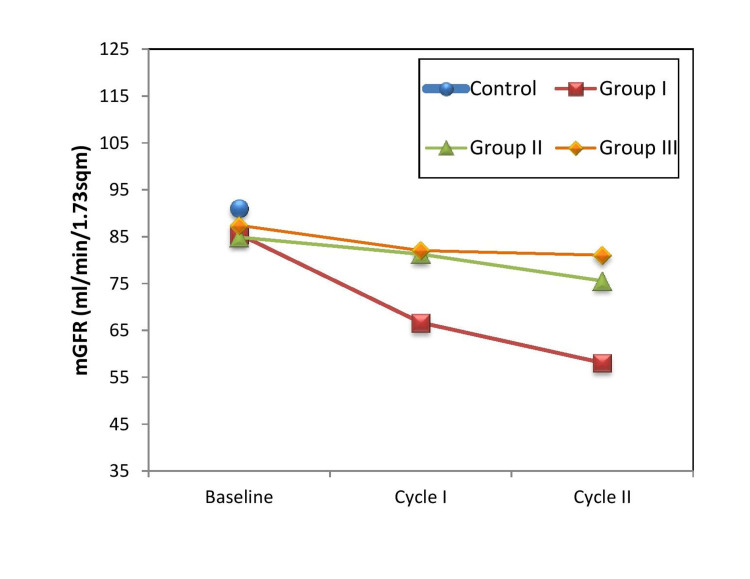
Graphical representation of mGFR (ml/min/1.73sqm) in control and three study groups of cancer patients The patients received chemotherapy *viz*. cisplatin, carboplatin, and oxaliplatin in respective groups.

Comparison of mean values according to cycles of chemotherapy between the groups shows that baseline magnitude among the control and study groups revealed no significant difference. Whereas in cycle I the data for GFR revealed that the maximum value was present in group III 82.05±13.92 and minimum value was in group I 66.66±22.40. Comparison between the groups showed that the GFR mean value in cycle I of group I was significantly lower (p<0.0001) than those of group II and group III (82.05±13.92). Similarly, on cycle II the mean value of group I (58.09±21.86) was significantly lower (p<0.000) than those in group II (75.55±16.49) and group III (81.02±12.98) (Figure 2).

The mGFR level in our patients decreased significantly after the first cycle of cisplatin-based chemotherapy (mGFR: 85.49 ± 20.83 vs 66.66 ± 22.40; n = 50; P < 0.001). Again, after the second cycle of chemotherapy, the mGFR decreased significantly, as compared to the pre-chemotherapy value (mGFR: 85.49 ± 20.83 vs, 58.09 ± 21.86; n =50 ; P < 0.001). Similarly, after the second cycle of carboplatin-based chemotherapy, the GFR decreased significantly, as compared to the pre-chemotherapy value (mGFR: 84.86 ± 10.33 vs, 75.55 ± 16.49; n =50 ; P < 0.001). While in oxaliplatin-based chemotherapy mGFR decreased in consequent cycles; however, the difference was not statistically significant (Table 3). 

**Table 3:** Comparison of mGFR (ml/min/1.73sqm) at baseline, cycle I and cycle II of chemotherapy within group. ** p < 0.001, n = 50 patients in each group

Groups	Baseline	Cycle I	Cycle II
Cisplatin Group I	85.49 ± 20.83	**66.66 ± 22.40	**58.09 ± 21.86
Carboplatin Group II	84.86 ± 10.33	81.27 ± 9.74	**75.55 ± 16.49
Oxyplatin Group III	87.38 ± 13.32	82.05 ± 13.92	81.02 ± 12.98

Accordingly, we compared the mGFR between the groups after the 1st and 2nd cycle of chemotherapy of the patients; mGFR decreased significantly in cisplatin-based chemotherapy as compared to carboplatin and oxaliplatin-based chemotherapy (Table 4).

**Table 4:** Comparison of mGFR (ml/min/1.73sqm) at baseline, cycle I and cycle II of chemotherapy between groups. **  p < 0.001, n=50 patients in each group

Cycles	Cisplatin Group I	Carboplatin Group II	Oxaliplatin Group III
Baseline	85.49 ± 20.83	84.86 ± 10.33	87.38 ± 13.32
Cycle I	**66.66 ± 22.40	81.27 ± 9.74	82.05 ± 13.92
Cycle II	**58.09 ± 21.86	75.55 ± 16.49	81.02 ± 12.98

## Discussion

Measurement of glomerular filtration rate (GFR) is necessary to know the state of renal functions, especially when serum urea and creatinine levels are normal. GFR estimation is a highly objective method to evaluate kidney functions. Earlier creatinine clearance was used for renal function evaluation which is more cumbersome and time-consuming. Later on it was replaced by the 51Cr-EDTA labeled test used to calculate GFR. At present 99mTc-DTPA renogram is widely used and Gate’s method of mGFR calculation is used [5]. In various study it was observed that platinum-based therapies have dose-limiting nephrotoxicity and especially cisplatin have highest level of toxicity to cause oliguria. In comparison to cisplatin, decreased urine volume is relatively less with carboplatin and it was attributed to a relatively lower drug accumulation in the renal tubular epithelium via the basement transport mechanism [6]. 

A decade ago cellular mechanism of cisplatin nephrotoxicity was explained and found that cisplatin preferentially accumulates in the cell S3 segment of renal proximal tubule and is toxified intracellularly by hydration. Its toxic manifestation occurs due to the inhibition of protein synthesis and binding to the -SH (sulfhydryl) group, leading to GSH (glutathione) depletion resulting in lipid peroxidation and mitochondrial damage [7]. In other studies it was reported that nephrotoxicity is an inherent adverse effect of certain anticancer drugs on vascular and parenchyma of the kidney [8]. The mechanism of cisplatin nephrotoxicity is thought to be renal tubular damage by uptake into the S3 segment of the proximal tubule through the organic cation transporter-2 and the copper transporter 1 [9]. 

Perhaps the frequency of renal insufficiency is underestimated in routine clinical practice because mostly serum creatinine measurements are done to know the renal insufficiency. Since serum creatinine is not interpreted together with the gender, age, and weight of the patient, it may not be an appropriate tool. GFR by radioisotope method can be an alternative pretreatment workup for cisplatin-based chemotherapy. Before infusion of cisplatin-based chemotherapy, it is safer to confirm estimated GFR (eGFR) by mGFR especially in elderly patients or in whom serum creatinine levels are borderline higher value [10]. 

In our study, age and gender distribution among all the groups was almost equal therefore we presume that all the body organs including kidney and bone marrow would be uniformly affected. However, no significant difference was observed in drug effects in terms of age and gender in our patients (Table 1). We calculated GFR using the 99mTc-DTPA method as we have a gamma camera in our institute. GFR was measured in ml/min/1.73sqm. In the cisplatin group there is a gradual fall of GFR from 85.49 ml/min/1.73sqm to 58.09 ml/min/1.73sqm at cycle II. In the carboplatin group it was 84.86ml/min/1.73sqm at baseline whereas cycle II was 75.5 ml/min/1.73sqm with SD ± 16.49. Whereas, in the oxaliplatin-treated group no significant falls were observed (Table 2). This show that cisplatin and carboplatin are more nephrotoxic and decreasing GFR is an important parameter to know the nephrotoxicity (Tables 3, 4).

The literature includes several studies on cisplatin nephrotoxicity, including the evaluation and prevention of cisplatin-induced nephrotoxicity. Tezcan et al. [11] documented that the GFR levels 6 weeks after completion of cisplatin-based chemotherapy were significantly lower than the pre-treatment value (GFR: 111.58 ± 9.28 vs 86.59 ± 8.20; P < 0.05). Similar to the present study, in Lauritsen et al. study, post-chemotherapy mGFR decreased in comparison to pre-treatment (p < 0.001). mGFR decreased median 9 mL/min/1.73 sqm after three cycles, 14 mL/min/1.73 sqm after four cycles, and 20 mL/min/1.73 sqm after 5+ cycles [12].

Our finding of decreased GFR in our patients corroborated with the observation of other studies where Sleijfer et al. observed a median decrease in GFR of 19.0% and they concluded that carboplatin cause considerable loss of renal function [13].

In a review of the comparative pharmacology of cisplatin and carboplatin it was concluded that patients with GFR less than 30 ml/min should not get cisplatin, whereas those with more than 50ml/min can get full doses and those who have GFR in between should get 30-60% of required dose [14]. 

In an animal study [15] in Wistar rats, it was observed that platinum compounds caused significant renal damage leading up to 84% decreased in GFR following cisplatin therapy. However, carboplatin caused a lesser degree of nephrotoxicity.

In a comparative study [16] it was observed that the creatinine clearance method and plasma clearance of 99mTc-DTPA method are equally sensitive methods to measure GFR and nephrotoxicity.

Calvert et al. [17] developed a formula to calculate carboplatin dose to avoid the development of nephrotoxicity. The formula is presented as total dose (mg) = target area under the plasma concentration-time curve (AUC) x [GFR+25]. The impaired renal function have higher than expected AUC for total platinum and to avoid untoward toxicity, it is necessary to reduce the dose of platins when treating patients with poor renal functions. As it was noticed that hematological toxicity was exaggerated in patients with poor renal function.

Observing the nephrotoxic effects of the platin group of drugs, we found that oxaliplatin is a much safer drug. It was well tolerated by cancer patients and also showed minimum side effects. However, the drug is quite expensive, which reduces its usage. Therefore, we recommend that in the general interest of cancer patients, the government should stand for the cost and proper measures should be taken to make it liberally available for the low socio-economic group of patients.

## Conclusions

Nephrotoxicity is the major side effect of platin drugs, and these side effects limit the use of these drugs. We strongly propose that further studies should be done to improve their therapeutic value and minimize toxicity by trying various cytoprotective agents. A trial could be done in collaboration with the pharmaceutical industry to change the chemistry of the platin drugs in order to increase the efficacy and reduce the level of toxicity.
